# The design of an Obstetric Telephone Triage Guideline (OTTG): a mixed method study

**DOI:** 10.1186/s12905-024-03076-1

**Published:** 2024-04-18

**Authors:** Farzaneh Rashidi, Masoumeh Simbar, Saeed Safari, Zahra Kiani

**Affiliations:** 1 Midwifery and Reproductive Health Research Center, School of Nursing and Midwifery, Shahid Beheshti University of Medical Sciences, Tehran, Iran; 2 Midwifery and Reproductive Health Research Center, Department of Midwifery and Reproductive Health, School of Nursing and Midwifery, Shahid Beheshti University of Medical Sciences, Tehran, Iran; 3 Department of Emergency Medicine, Shohadaye Tajrish Hospital, Shahid Beheshti University of Medical Sciences, Tehran, Iran; 4 Midwifery and Reproductive Health Research Center, School of Nursing and Midwifery, Shahid Beheshti University of Medical Sciences, Tehran, Iran

**Keywords:** Triage, Obstetrics, Obstetric triage, Gynecology, Clinical guidelines

## Abstract

**Background:**

Clarifying the dimensions and characteristics of obstetric telephone triage is important in improving the quality of services in the health system because researchers can evaluate the effectiveness of treatment, care and diagnostic measures in the form of obstetric telephone triage by developing a guideline. Therefore, this study aimed to design an Obstetric Telephone Triage Guideline (OTTG) using a mixed-method study.

**Methods:**

The present study was carried out using an exploratory sequential mixed method study in two qualitative and quantitative phases. An inductive-deductive approach was also used to determine the concept of obstetric telephone triage. In this respect, a qualitative study and a literature review were used in the inductive and deductive stages, respectively. Moreover, the validity of the developed guideline was confirmed based on experts’ opinions and results of the AGREE II tool.

**Results:**

The guideline included the items for evaluating the severity of obstetric symptoms at five levels including “critical”, “urgent”, “less urgent”, “no urgent”, and “recommendations”. The validity of the guideline was approved at 96%, 95%, 97%, 95%, 93%, and 100% for six dimensions of AGREE II including scope and purpose, stakeholder involvement, the rigor of development, clarity of presentation, applicability, and editorial independence, respectively.

**Conclusion:**

The OTTG is a clinically comprehensive, easy-to-use, practical, and valid tool. This guideline is a standardized tool for evaluating the severity of symptoms and determining the urgency for obstetrics triage services. By using this integrated and uniform guideline, personal biases can be avoided, leading to improved performance and ensuring that patients are not overlooked. Additionally, the use of OTTG promotes independent decision-making and reduces errors in triage decision-making.

## Introduction

Hospitals receive 20 to 30 phone calls from pregnant women per day. During these calls, healthcare providers use their obstetric knowledge and experiences to determine the severity of the problem and the urgency of a physical consultation with an obstetrician [1–3]. Obstetric triage services are usually provided physically (face-to-face), however, in many Western societies, women typically make an initial phone call to inquire about the need for counseling or a visit to the emergency room [4–9]. Telephone triage is a service delivery system that is currently not performed uniformly due to the lack of specific instructions [1–3].

While most existing guidelines for obstetric triage are based on physical triage. However, telephone triage has many positive aspects such as efficacy and uniformity. However, there are some challenges due to the lack of clinical perspective during the telephone call and the lack of specific diagnostic information [10].

Obstetric physical triage is an independent and efficient unit [11, 12] with versatile dimensions such as parturient admission, evaluation of fetal well-being, as well as acute obstetric and gynecological emergencies [13]. The use of triage is important throughout the pregnancy and labor stages. Triage ensures appropriate care based on the clinical priorities of the patient and the effective use of resources [14, 15]. However, some problems of obstetric triage include patient dissatisfaction and prolonged waiting times [16–18]. Prolonged waiting time results in leaving patients without examination, delayed delivery of necessary care and treatment, patient dissatisfaction, and increased mortality and morbidity [19, 20]. On the other hand, reducing the waiting time decreases the duration of hospital stay, lowers the treatment cost, and saves hospital resources [21].

Studies show that triage standardization favorably affects its efficiency and safety [17]. The telephone is a 24-hour means of communication between patients and health care providers [22], and the importance of the telephone answering system has been specified [23].

Some of the advantages of telephone triage include increased access to services, reduced waiting time to receive services, avoidance of unnecessary referrals, and long and expensive trips [24, 25].

Understanding the correct telephone triage approach by obstetric triage personnel is extremely important since improper communication leads to untimely acceptance of pregnant women and incorrect decisions [26].

The results of a review study showed that there is no agreement on the existence of a standard system in the field of obstetric triage [17]. Bailey et al. (2018) conducted a review study to investigate obstetric telephone triage. The results showed that more research is needed to examine midwives’ perceptions of their role in obstetric telephone triage, reviewing obstetric telephone triage processes and tools [27]. Engeltjes et al. (2020) conducted a Delphi study in the Netherlands to design obstetric telephone triage guidelines. The telephone obstetric triage guidelines were designed based on the physical obstetric triage system ROTS with five prioritization categories, which 91.9% of professional users declared complete, good, and user-friendly [28]. Limited studies have focused on the design and psychometrics of obstetric telephone triage guidelines [27], whereas the design of obstetric telephone triage guidelines can strengthen obstetric performance [29].

Given the importance of clarifying the dimensions and characteristics of obstetric telephone triage in improving the quality of services in the health system, researchers can evaluate the effectiveness of treatment care and diagnostic measures in the form of obstetric telephone triage by using this guideline. In addition, different health service providers and managers can use the results of these assessments to improve the quality of obstetric telephone triage. With this background in mind, the present study aimed to develop an obstetric telephone triage guideline.

## Methods

**Fig. 1:**
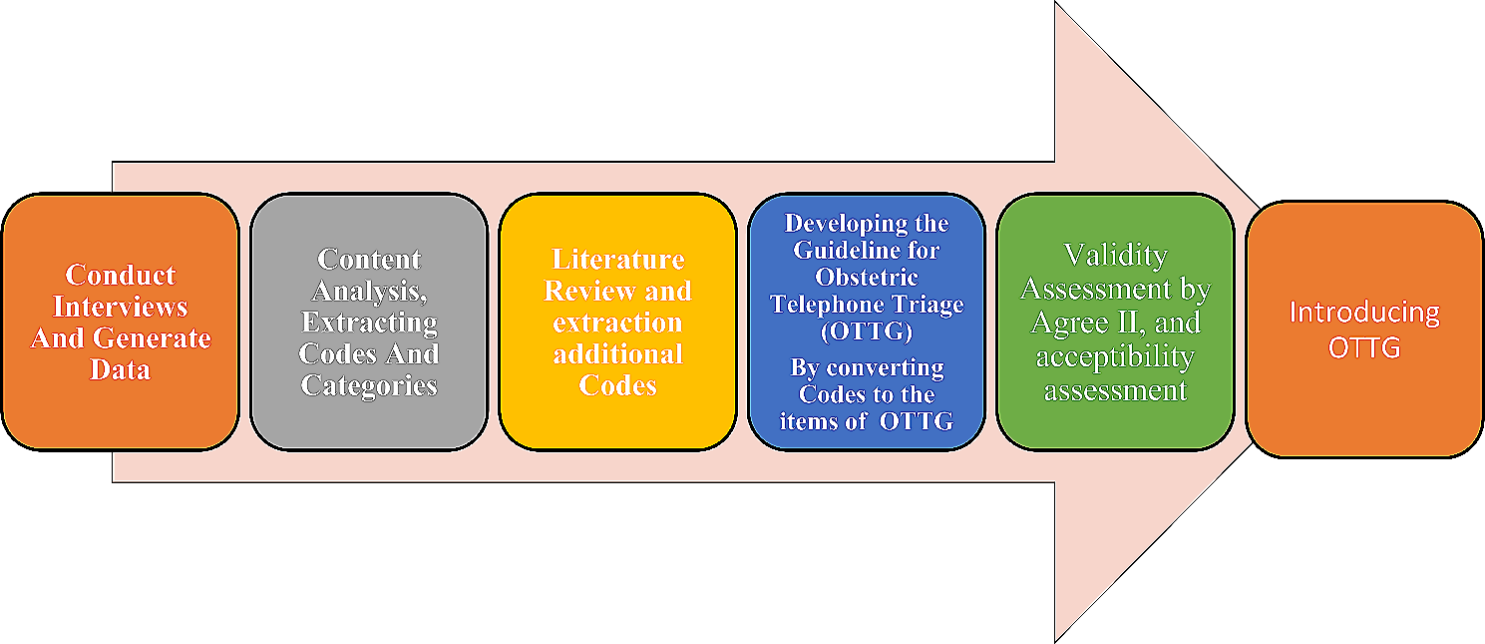
The procedure of the study to develop and validation of Obstetric Telephone Triage Guideline (OTTG)

This was an exploratory sequential mixed method study in two qualitative and quantitative phases to develop and validity assessments of the guideline an inductive-deductive approach was performed to explain the concept of obstetric telephone triage. In this respect, a qualitative study and a literature review were carried out to extract the codes and convert them to the items of the OTTG. After item generation and developing the first draft of OTTG, its validity was assessed by the experts using Appraisal of Guidelines for Research and Evaluation II (AGREE II). Figure 1 shows the procedure of the study for OTTG development and validation.

The details for the procedure of the study are as follows:

### Qualitative phase

This qualitative study with a content analysis approach was performed as the first part of a multi-stage large study [30–32].

The participants with maximum diversity and different educational levels, fields, and specialties related to obstetric triage were interviewed. The mean call volume was 400 calls per month.

The obstetric triage personnel were recruited from medical educational hospitals with obstetric and telephone triage services, specifically from Shahid Beheshti University of Medical Sciences in Tehran-Iran.

The inclusion criteria for obstetric triage personnel were: the ability to express experiences, and willingness to participate in the study, having at least 6 months of work experience in the obstetric department, and being familiar with telephone triage. The exclusion criteria included: refusing to continue the interview.

The participants were recruited using the purposive sampling method that continued until data saturation. The semi-structured individual interviews were performed to explain the participants’ perceptions and work experiences about telephone triage. The interviews were conducted by the first author of the research, and recorded and immediately transcribed verbatim. Following receiving permission to do the research in the setting, the objectives were explained to the participants of the study before the interviews.

Afterward, informed consent was obtained from all participants and they were ensured of the confidentiality of the information. Moreover, participation in the study was voluntary. The participants were asked to add anything left about the subject at the end of the interviews. Ultimately, the researcher referred to the possibility of future interviews in addition to appreciating the cooperation of the participants. Data analysis was conducted using Granheim and Lundman’s method, which was performed simultaneously with data collection [33]. The interviews were transcribed immediately after each interview and completed on the same day. The average, duration of the interviews was 28.52 min (with a duration between 20 and 49 min). Based on the participants’ viewpoints, the most significant factors influencing obstetric telephone triage were categorized into two main categories: common complaints (27 codes) and communication (8 codes). The initial guideline framework, number of levels, and criteria for evaluating maternal and fetal health were developed based on the findings of a qualitative study.

In the deductive approach and using a literature review, a comprehensive search was conducted on databases such as Scopus, PubMed, Embase, ProQuest, Web of Science, Cochrane, Science Direct, and Google Scholar, using keywords such as pregnancy, obstetric, triage guideline, system, index, tool, questionnaire, scale, telephone, midwifery, and maternity. A total of 26 articles were included in the study, and items related to obstetric telephone triage not existing in the pool of items obtained from the qualitative stage were added to the main items.

### Quantitative phase (validity assessment)

The AGREE II tool was used to assess the internal validity of the guideline. The primary draft of the guideline was sent via email to 10 experts in questionnaire development, emergency medicine, reproductive health, obstetrics, and telephone triage (different from the participants in the qualitative phase) for their feedback on the decision-making process for the risk classification based on maternal and fetal criteria and symptoms, the severity of the emergency, patient arrival time at the hospital.

AGREE II encompasses 23 key criteria in six domains scope and purpose (criteria 1–3), stakeholder involvement (criteria 4–7), rigor of development (criteria 8–14), clarity of presentation (criteria 15–17), applicability (criteria 18–21), and editorial independence (criteria 22 and 23) [27].

The score of each domain was calculated by adding the score given to the criteria and after total score calculation, it was standardized by converting to a 0-100 score range based on the following formula: X-Min Score / Max-Min Score) × 100. The guideline remained in the study in case of earning a score above 60% in each area [34].

To assess the acceptability of the guideline, it was sent again to the same 10 experts via email who rated the final guideline in terms of feasibility and applicability (extremely high = 4, high = 3, low = 2, and very low = 1), being scientific (completely agree = 4 to completely disagree = 1), and importance (completely agree = 4 to completely disagree = 1). Finally, data analysis was performed, and validity was confirmed with 70% consensus about the acceptable utility of the guideline [35].

## Finding

The participants were 21 obstetric triage personnel and the key informants, including emergency medicine specialists, clients, gynecologists, and reproductive health specialists. The average work experience of obstetric triage personnel was 13.6 years (work experience of 3 to 28 years).

Based on the results of the qualitative phase, 124 primary codes and 35 integrated codes were extracted through the semi-structured interviews.

According to the participants’ opinion, the most significant factors influencing the use of obstetric telephone triage services were in two main categories: common complaints (27 codes) and communication (8 codes).

The common complaints category includes items such as abdominal pain, hemorrhage, hypertension, postpartum concerns, membrane rupture, suspicion of labor onset, illness/other, and decreased fetal movements. The communication category includes items such as last menstrual period, age, gestational age, number of pregnancies and parity, and obstetric and medical history. These items were included in the obstetric telephone triage guidelines. The extracted items from the qualitative interviews are listed in Table 1.

**Table 1:** The categories and items extracted from the qualitative interview

Category	Items Extracted from The Qualitative Interview
**Common Complaints**	Breathing Disorder and Consciousness
	Convulsions
	Fever
	Lethargy
	Blurred vision
	Trauma/abdominal blow/fall
	Uterine contractions (labor pain/contraction/tightening of the abdomen and uterus)
	Heavy vaginal bleeding/clotting
	Severe headache
	Dyspnea/chest pain less than 12 h
	Epigastric Pain with Nausea and Upper Abdominal Pain/Stomach Pain
	The Feeling of The Umbilical Cord Protruding by The Mother
	Absence of fetal movement
	Vaginal discharge
	Vaginal colored discharge
	Swelling and one-sided leg and thigh pain
	Pruritus
	Nausea
	Diarrhea/constipation
	Problems in the Extremities Limb
	Back pain / pelvic girdle pain
	Stress and anxiety/emotional problems in the last 24 h
	Dysuria
	High blood pressure
	Hemorrhoids
	Oral and dental problems (oral abscess and gingivitis)
	Body bruising
**Communication**	Age
	Last menstrual of period
	Estimated date of delivery
	Gestational age
	Number Of Pregnancies And parity
	Information about medications
	History of obstetrics and medicine

Ten items were also extracted from the review of the searched articles. In the deductive approach, otherwise, the items related to obstetric telephone triage, which were not identified in the qualitative phase, were then added to the main items. These additional items include headache with aura, headache without aura [36], severe and continuous pain after 24 weeks of gestation [28], lack of fetal movements before 20 weeks [28], lack of fetal movements between 20 and 24 weeks, pain and gestational age between 24 and 37 weeks [28], confirmed uterine pregnancy by ultrasound or unconfirmed pregnancy by ultrasound between 5 and 16 weeks [37] and mild vaginal bleeding [38, 39], dyspnea lasting less than 24 h and mild diarrhea before 16 weeks [28, 40].

Finally, the items extracted from the qualitative study (using an inductive approach) were integrated with the items extracted from the literature review (using a deductive approach) results and initial draft of the obstetric telephone triage guideline was created.

**Fig. 2:**
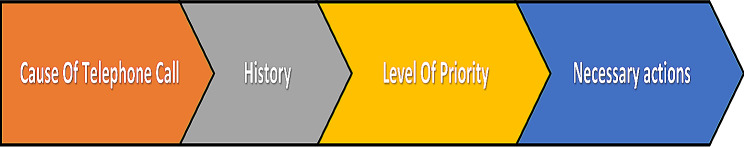
The components of obstetric telephone triage guideline

Figure 2 the components of obstetric telephone triage guidelines.

**Fig. 3:**
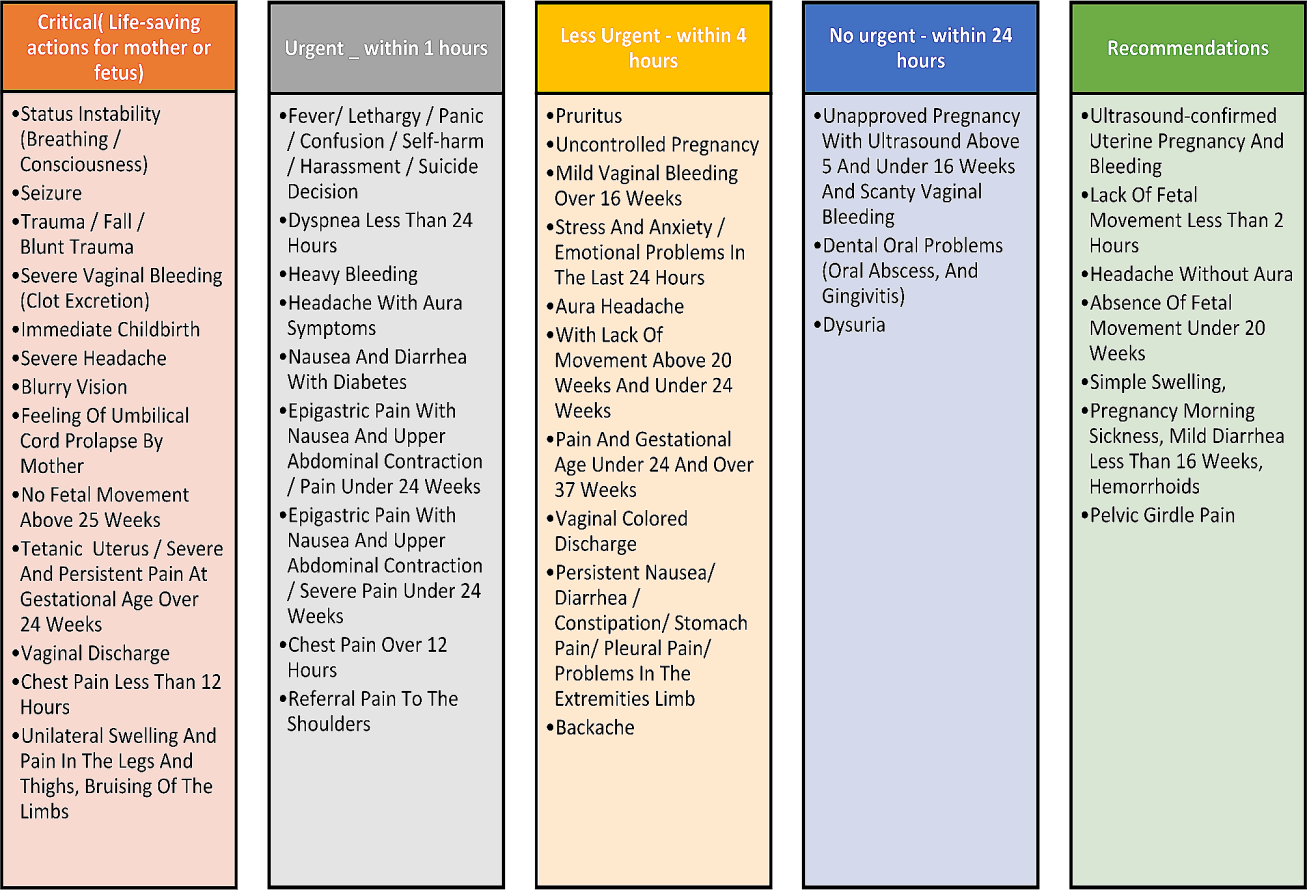
Levels of priority based on the obstetric telephone triage guideline

The guideline was then reviewed by experts, who prioritized the recommendations into five levels: critical, urgent, less urgent, no urgent, and recommendation. The critical level included life-saving measures for both the mother and fetus, while the other levels consisted of hospital referral within one hour, four hours, and 24 h, as well as the need for a consultation (Fig. 3).

The obstetric telephone triage guidelines included multiple levels and evaluation criteria for both the mother and fetus and were once again provided to experts for validation.

Table 2 shows the validity and quality of the developed guideline based on the AGREE II tool (Table 2). According to the experts’s opinion, the score obtained in all domains of the AGREE II tool was above 60%, so the designed guideline has a content validity.

**Table 2:** The percent agreement with the domains in the AGREE instrument by 10 experts

Domains	Percent(%)
Scope and purpose	96
Stakeholder involvement	95
Rigor of development	97
Expressiveness and presentation	95
Applicability	93
Editorial independence	100

The final validation of the guideline was carried out based on the opinions of experts in terms of applicability (93%), scientific (90%), and importance (100%).

## Discussion and recommendations

The present study introduces an understandable, applicable, and valid guideline for obstetric telephone triage. This guideline is the first obstetric telephone triage guideline designed in Iran, which is based on the modified Emergency Severity Index (ESI) obstetric triage [41]. This guideline helps to perform obstetric telephone triage based on cause, the severity of symptoms, and treatment for pregnant women in 5 levels including Critical (life-saving measures for mother or fetus), Urgent (visit the hospital within an hour), Less Urgent (Refer to the hospital within four hours), No urgent (refer to the hospital within twenty-four hours) and Recommendations (need to consult) [41]. In a study conducted in the United States of America to develop a telephone triage of candidiasis diagnosis, Hoffstetter et al. (2012) showed that it is difficult to diagnose candidiasis using symptoms and self-report through telephone triage as well as telephone treatment in symptomatic women. However, a telephone symptom guide for the diagnosis of candidiasis increased the accuracy of treatment by telephone triage [42]. While obstetric telephone triage guidelines are intended to guide triage personnel, they should not reduce their decision-making ability or professional responsibility and should be consistent with the patient’s professional insights and preferences.

Based on a survey, providing friendly advice in telephone triage leads to mothers’ reassurance and prevention of premature delivery for childbirth [43]. In a study, Huibers et al. reported that the majority of calls responded to by triage personnel were non-urgent [44]. In a study, pregnant women often make several calls for pregnancy and childbirth counseling. Triage personnel often provide different responses that are not documented, which leads to safety problems for the caller. Therefore, it is important to develop an evidence-based process for telephone triage [45]. In a study by Wahlberg et al., triage personnel reported callers’ anger, loud voice, and disrespectful behavior. In addition, they barely listened to the triage personnel’s guidance and were ultimately dissatisfied with the call. An inaccurate understanding of the situation or exaggeration of the event disrupts triage personnel’s judgment and causes loss of information [46]. Obstetric telephone triage is a complex multifaceted process affected by different internal and external factors [27]. Having a unified national guideline on telephone triage can reduce weaknesses in management and planning.

The results of the present study showed the validity of 93%, 90%, and 100%, respecting the guideline’s applicability, scientific, and importance, respectively. In a study by Engeltjes et al., an obstetric telephone triage guideline was designed based on the Rotterdam Obstetric Triage System (ROTS) in the Netherlands. The guideline included the examination of emergency obstetric symptoms at five levels. Their results showed that 91.9% of the experts considered the tool to be complete, accurate, and user-friendly, and 98.4% of them expressed that the tool was ready to be used [28]. The results of the studies showed that the validity of The Maternal Fetal Triage Index (MFTI), the Perinatal Emergency Team Response Assessment (PETRA), and the Swiss Emergency Triage Scale (SETS), all three obstetric triage systems, were 72.9%, 80%, and 78.4%, respectively [6, 8, 47].

The validation was performed using different methods for triage guidelines. The results showed that external validity is one of the most valid methods [15]. In maternal care, developing valid obstetric triage guidelines is critical [48, 49], and that also requires training packages [50]. There is limited evidence on the implementation and evaluation of such a system [51]. A successful triage unit follows a consistent policy. All instructions should be for the management of all clients for various reasons. If providers fail to follow the guidelines or standards, difficulties in fulfilling responsibilities and risks for clients may occur [52]. The guidelines allow all triage personnel to quickly evaluate and provide services to clients. Coordinated use of protocols, guidelines, and team training are effective ways to improve the quality of the services. In all cases, the use of guidelines and timely evaluation enables team action and coordination to ensure appropriate results [53].

One of the major limitations of the present study was the lack of access to obstetric telephone triage guidelines of different countries with various healthcare systems in the world. Another limitation of the present study was the lack of consideration of the patient’s voice and cultural factors.

## Conclusion

In this study, we examined various dimensions of obstetric telephone triage. Our findings indicate that the obstetric telephone triage guideline is clinically comprehensive, easy to understand, practical, and valid. This guideline serves as a standardized tool for evaluating the severity of symptoms and determining the urgency of obstetric care for triage personnel. By following this integrated and uniform guideline, personal biases can be avoided, leading to improved performance and ensuring that patients are not overlooked. Additionally, the use of an integrated obstetric telephone triage guideline promotes independent decision-making and reduces errors in triage decision-making.

## Data Availability

The data that support the findings of this study are available from Masoumeh Simbar but restrictions apply to the availability of these data, which were used under license for the current study, and so are not publicly available. Data are however available from the authors upon reasonable request and with permission of Shahid Beheshti University of Medical Sciences.

## Abbreviations

AGREE: Appraisal of Guidelines Research and Evaluation
ROTS: The Rotterdam Obstetric Triage System
OTTG: Obstetric Telephone Triage Guideline
MFTI: The Maternal Fetal Triage Index
PETRA: Perinatal Emergency Team Response Assessment
SETS: Swiss Emergency Triage Scale
